# Lung-derived soluble factors support stemness/plasticity and metastatic behaviour of breast cancer cells via the FGF2-DACH1 axis

**DOI:** 10.1007/s10585-024-10284-4

**Published:** 2024-04-06

**Authors:** Vasudeva Bhat, Matthew Piaseczny, David Goodale, Urvi Patel, Ashkan Sadri, Alison L. Allan

**Affiliations:** 1 London Regional Cancer Program, London Health Science Centre, London, ON N6A 5W9 Canada; 2 Department of Anatomy & Cell Biology, Schulich School of Medicine and Dentistry, Western University, London, ON N6A 5C1 Canada; 3 Department of Oncology, Schulich School of Medicine and Dentistry, Western University, London, ON N6A 5W9 Canada

**Keywords:** Breast cancer, Lung metastasis, Lung microenvironment, Stemness/plasticity, Fibroblast growth factor 2 (FGF2), Dachshund homolog 1 (DACH1)

## Abstract

Patients with triple-negative breast cancer (TNBC) have an increased propensity to develop lung metastasis. Our previous studies demonstrated that stem-like ALDH^hi^CD44^+^ breast cancer cells interact with lung-derived soluble factors, resulting in enhanced migration and lung metastasis particularly in TNBC models. We have also observed that the presence of a primary TNBC tumor can ‘prime’ the lung microenvironment in preparation for metastasis. In this study, we hypothesized that soluble lung-derived factors secreted in the presence of a primary TNBC tumor can influence stemness/plasticity of breast cancer cells. Using an ex vivo pulmonary metastasis assay (PuMA), we observed that the lung microenvironment supports colonization and growth of ALDH^hi^CD44^+^ TNBC cells, potentially via interactions with lung-derived FGF2. Exposure of TNBC cells to lung-conditioned media (LCM) generated from mice bearing TNBC primary tumors (tbLCM) significantly enhanced the proportion of ALDH^hi^CD44^+^ cells compared to control or LCM from tumor-naïve mice (tnLCM). Further analysis using a human cancer stem cell qPCR array revealed that, relative to tnLCM or control, exposure of TNBC cells to tbLCM leads to downregulation of the transcription factor and putative tumor suppressor Dachshund homolog 1 (DACH1), a downstream regulator of FGF2. In addition, inhibition of DACH1 using siRNA or treatment with recombinant FGF2 enhanced the ALDH^hi^CD44^+^ phenotype. Taken together, our findings suggest that the FGF2-DACH1 signaling axis supports stemness/plasticity of TNBC cells in the lung microenvironment and lays the foundation for future evaluation of FGF2 as a potential novel therapeutic target for treatment or prevention of breast cancer metastasis to the lung.

**Supplementary Information:**

The online version contains supplementary material available at 10.1007/s10585-024-10284-4.

## Introduction

Despite advances in early diagnosis and improved treatment approaches, breast cancer remains a leading cause of breast cancer diagnosis and death in women, with over 90% of breast mortality occurring as a result of metastasis [1, 2]. Breast cancer is a heterogenous disease, and in the clinical setting breast cancer is primarily classified into four different molecular subtypes. These include luminal A (ER^+^/PR^+^, Ki67^−^), luminal B (ER^+^/PR^+^, HER2^+/−^, Ki67^+^), HER2-enriched (ER^−^, PR^−^, HER2^+^) and triple negative (TN; ER^−^, PR^−^, HER2^−^) subtypes [3]. Among these subtypes, TN breast cancer (TNBC) is very aggressive and highly prone to metastasis, resulting in poor survival outcomes [4, 5]. Patients with TNBC are mainly treated with systemic chemotherapy due to limited targeted therapy options, often resulting in the development of resistance to non-specific chemotherapy, particularly in the metastatic setting [1, 6]. Therefore, gaining a greater understanding the cellular and molecular processes that drive breast cancer metastasis is important for developing new targeted therapy approaches to prevent or treat metastatic disease.

The metastatic process involves the spread of tumor cells from the primary breast tumor via the circulatory or lymphatic system to distant organs such as the lung, liver, brain and bone where they can form clinically detectable secondary lesions [7, 8]. Successful metastasis depends on the ability of disseminated cells to both initiate tumor growth at the secondary site as well as maintain growth to progress to a clinically detectable macroscopic tumor. However, only a small number of cells survive and successfully form new tumors at distant sites [9]. This suggests that both cancer cell-intrinsic factors as well as cell-extrinsic features of the target organ play a pivotal role in metastasis. The seed and soil hypothesis proposed by Stephen Paget clearly demonstrates the importance of cancer cells with survival/growth abilities (the seed) and the soluble and insoluble components of metastatic microenvironment (the soil) for successful metastasis [10].

The lung is one of the most common and deadliest sites for breast cancer metastasis, especially in TNBC patients [11, 12]. The increased propensity of TNBC cells to metastasize to the lung is often due to interactions between breast cancer cells and the lung microenvironment [13]. In particular, our previous studies have shown that TNBC cells with high aldehyde dehydrogenase activity (ALDH^hi^) and expression of cluster of differentiation 44 (CD44^+^) (considered a “stem-like” ALDH^hi^CD44^+^ phenotype [14]) display enhanced metastatic potential and higher propensity to migrate and metastasize to lungs [15], potentially via interacting with soluble lung-derived factors including fibroblast growth factor 2 (FGF2) [16]. We have also observed that that the presence of a primary TNBC can preferentially ‘prime’ both the stromal and soluble components of the lung microenvironment in preparation for metastasis [17]. However, the mechanisms underlying the successful growth and metastatic colonization of stem-like ALDH^hi^CD44^+^ TNBC cells in the primed lung microenvironment remain poorly understood.

In the current study, we hypothesized that lung-derived soluble factors secreted in the presence of a TNBC primary tumor influence stemness/plasticity and metastatic behavior of stem-like ALDH^hi^CD44^+^ breast cancer cells. Our findings suggest that the lung microenvironment favors the growth and colonization of stem-like ALDH^hi^CD44^+^ breast cancer cells over non-stem-like ALDH^lo^CD44^−^ cells and that blockade of FGF2 in the lung results in decreased growth and colonization of TNBC potentially by targeting stemness/plasticity. We also demonstrate that the transcription factor and putative tumor suppressor *DACH1* (a downstream target of FGF2) is significantly downregulated by lung-derived soluble factors secreted in the presence of primary TNBC tumor, and that inhibition of *DACH1* through siRNA or FGF2 treatment increases acquisition of a stem-like ALDH^hi^CD44^+^ breast cancer phenotype. Together, these results suggest that lung-derived FGF2 may influence stemness/plasticity and metastatic behavior of breast cancer cells by inhibiting *DACH1* expression.

## Materials and methods

### Cell culture and reagents

Human MDA-MB-231 TNBC cells were obtained from American Type Culture Collection (ATCC, Manassas, VA, USA) and maintained in Dulbecco’s modified Eagle’s medium (DMEM)/F12 + 10% fetal bovine serum (FBS). Human SUM149 and SUM159 TNBC cells were obtained from Asterand Inc (Detroit, MI, USA) and maintained in HAMS: F12 + 5% FBS supplemented with 5 µg/ml insulin, 1 µg/ml hydrocortisone and 1mM HEPES. All the cell lines were authenticated via third party testing (IDEXX BioAnalytic, Columbia MO, USA; 08/2021). Breast cancer cell lines expressing red fluorescent protein were generated by transducing lentivirus containing either a tdTomato (MDA-MB-231 and SUM149) or mCherry (SUM159) respectively and the cultures were maintained using puromycin as a selection marker. For siRNA experiments, TNBC cells (5000 cells/cm^2^) were plated in respective growth media, and 16 h post plating were transfected with 120pmol of non-targeting scrambled and *DACH1* targeting siRNA (Dharmacon, Lafayette, CO, USA) using RNAiMax (Invitrogen). Media was changed to regular growth media 6 h post transfection, and cultures were maintained until experimental end point. Knockdown efficiency was measured at 48 h and 72 h post transfection using RT-qPCR and immunoblotting as described below.

### Fluorescence activated cell sorting (FACS) and flow cytometry analysis

For FACS isolation of breast cancer subsets, single cell suspensions were prepared from mCherry expressing SUM149, SUM159 and tdTomato expressing MDA-MB-231 human breast cancer cells. Approximately 1 × 10^6^ cells were incubated with ALDEFLUOR™ substrate (Stemcell Technologies, Vancouver, BC, Canada) for 45 min at 37 °C. A control sample was also prepared in which the specific ALDH inhibitor diethylaminobenzaldehyde (DEAB) was co-incubated with ALDEFLUOR™. These cells were subsequently labelled with anti-human CD44 conjugated to Allophycocyanin (Clone G44-26, BD Bioscience, Mississauga, Canada) and 7-Aminoactinomycin (7AAD) (Mississauga, Canada) to exclude dead cells. Subsets of breast cancer cells including ALDH^hi^CD44^+^ and ALDH^low^CD44^−^ were isolated using FACS sorter (FACSAria II, BD Biosciences) in sterile phosphate buffer saline (PBS) containing 2% FBS on ice using the gating strategy shown in *Supplemental Figure **S1*. For analysis of ALDH^hi^CD44^+^ phenotype in response to different experimental conditions, cells were harvested, washed, resuspended in Aldeflour buffer, and incubated with Aldeflour substrate for 45 min as per the manufacturer’s instructions. Cells were subsequently labelled with anti-human CD44 conjugated to R-phycoerythrin (Clone G44-26, BD Bioscience, Mississauga, Canada) and 7AAD prior to analysis on a Beckman Coulter FC500 flow cytometer. Resulting data was analyzed using FlowJo software (BD Biosciences). For FGF2 blocking experiments, BM, tnLCM and tbLCM were pre-incubated with or without 1 mg/1 ml of neutralizing monoclonal FGF2 antibody (clone bFM-1; EMD Millipore, Massachusetts, USA) for 30 min at room temperature. SUM159 breast cancer cells were then exposed for 72 h and changes in the ALDH^hi^CD44^+^ phenotype were assessed via flow cytometry.

### Ex vivo pulmonary metastasis assay

Animal experiments were carried out in accordance with the Canadian Council of Animal Care under a protocol approved by the University of Western Ontario Animal Cancer Committee (2021-076). Unsorted and FACS sorted subsets (ALDH^hi^CD44^+^ and ALDH^lo^CD44^−^) of human TNBC cells were resuspended in 1X sterile PBS at a concentration of 5 × 10^6^ cells/mL and 100 µL (5 × 10^5^ cells) of unsorted and FACS sorted cells population were injected into the lateral tail vein of 6–8 week-old female nude mice (*n* = 3 mice/group). Fifteen minutes post injection, lungs were isolated and subjected to a ex vivo pulmonary metastasis assay (PuMA) as described previously [18, 19] and shown in *Supplemental Figure **S2**A*. Using sterile surgical conditions, the trachea was snipped and cannulated with an 18G blunt needle. Lungs were infused under gravitational pressure with 1.2 mL of equal amounts of well-mixed lung media/low melting agarose solution (0.6%, 40 °C). The trachea, lungs, and heart were carefully removed *en bloc* and immediately placed in ice-cold PBS containing 100U/ml penicillin and 100 µg/ml streptomycin and stored at 4 °C for 20 min to solidify the media/agarose solution within the lung. Transverse lung Sects. (1–2 mm in thickness) were cut from each lobe using a scalpel blade. Lung sections were carefully placed on a single sterile piece of Gelfoam (~ 1 cm x 1 cm) that had been preincubated for 1–2 hours in a 6 well plate with M199 media containing 1.0 µg/mL bovine insulin, 0.1 µg/mL hydrocortisone, 01 µg/mL retinyl acetate, 100U/mL penicillin, 100 µg/mL streptomycin and 7.5% sodium bicarbonate. Lung sections cultured in the ex vivo PuMA (days 0, 7, 14 and 21 post injection) were fixed overnight in 10% buffered formalin phosphate + 25% sucrose (w/v) to preserve fluorescent signal. The following day, lung sections were rinsed with PBS three times and placed on a 35 mm glass bottom dish. Images were acquired using an upright Nikon A1R confocal microscope at 10X objective, with a 591 nm emission laser, pin hole set to 10. Three separate lung sections were imaged per timepoint with five images taken per lung section. To assess growth and colonization of breast cancer cells, mean fluorescent area per field of view (FOV) for each lung section (µm^2^) was measured using ImageJ software (NIH, Bethesda, WA, USA). Data were normalized to 1000µm^2^ at day 0 to account for variability in cellular delivery during tail-vein injection. Metastatic progression was assessed by calculating the percentage of colonies per image as either single cells (≤ 50 μm), micrometastatic lesions (100–400 μm) or macrometastatic lesions (> 400 μm) according to measured diameter. For PuMA assays involving neutralization of FGF2, lungs seeded with breast cancer cells were infused with either normal agarose or agarose containing neutralizing monoclonal FGF2 antibody (1:200, clone bFM-1; EMD Millipore, Massachusetts, USA) prior to following the reminder of the PuMA protocol.

### Generation of lung-conditioned media

Human SUM159 TNBC cells were suspended in 1X phosphate buffer saline (PBS) at a concentration of 1 × 10^7^ cells/mL and 100µL of cell suspension (1 × 10^6^) were injected into the mammary fat pad (MFP) of 6–8 week old female nude mice (Athymic Nude-Foxn1nu; Envigo, Mississauga, ON, Canada) as previously described [20]. Primary tumor growth was longitudinally assessed weekly using digital caliper measurements in 2 perpendicular dimensions and was calculated using the formula: volume = 0.52 X (width^2^) X (length). Mice bearing SUM159 tumors were sacrificed 4 weeks post MFP injections. Age-matched tumor naïve mice were used as controls. Tumor naïve and tumor-bearing mice were euthanized, and lung-conditioned media (LCM) were generated essentially as previously described [16, 17]. Brief, lungs were aseptically harvested, washed, and kept in sterile PBS on ice. Lungs were weight-normalized by resuspending 4:1 media to tissue (vol/wt) ratio in DMEM: F12 supplemented with Mito^+^ serum extender (BD Biosciences, Mississauga, Canada) and cocktail of penicillin (50U/mL) + streptomycin (50U/mL) (Invitrogen). Lungs were minced into ~ 1 mm^3^ fragments and cultured for 24 h prior to collecting LCM. Conditioned media was then filter sterilized using 0.22 μm filters to remove cellular debris and stored at −80 °C until use. To account for mouse-to-mouse variability, LCM from multiple mice were pooled prior to use in experimental studies. The concentration of soluble FGF2 in base media (BM) control, tumor naïve (tnLCM) and tumor-bearing (tbLCM) lung conditioned media was measured using a Mouse bFGF ELISA kit (RayBiotech, USA) according to the manufacturer’s instructions.

### Sphere-forming assays

Human SUM159 TNBC cells were exposed to base media, tnLCM or tbLCM for 72 h. Cells were then trypsinized and 1000 viable cells were resuspended in mammosphere media containing DMEM: F12, 5 µg/ml insulin, 20ng/ml EGF, 0.04% BSA, 1X B27, 10ng/ml bFGF and seeded onto a 96-well ultra-low attachment plates. Samples were monitored for sphere formation over a period of 21 days. Spheres were imaged and counted using Olympus 1 × 70 microscope. Sphere formation efficiency was calculated by dividing total number for spheres formed in each well by the number of cells seeded x 100.

### Quantitative real-time PCR human cancer stem cell® array and RT-qPCR analysis

Total RNA was extracted from human SUM159 TNBC cells exposed to tnLCM and tbLCM using the TRIzol™ (Invitrogen) RNA purification method according to manufacturer’s instructions. Subsequently, cDNA was synthesized using RT2 First Strand kit (Cat. No. 330,401, Qiagen, Germany) and loaded onto 96-well RT^2^ Profiler™ PCR array Human Cancer Stem Cell arrays (Cat. No. PAHS-176ZC, Qiagen). Changes in cycle threshold (*Ct*) values were calculated using Qiagen’s online Data Analysis Centre. All transcript levels were normalized to the internal GAPDH control. RAW and normalized data is available in *Supplemental Table **S1*. For validation of Human Cancer Stem Cell® Array results and knockdown of *DACH1*, total RNA was extracted using TRIzol™ (Invitrogen) reagent and 1 µg of RNA was used to generate complementary DNA using Superscript IV VILO Master Mix (Invitrogen). Changes in transcript levels was quantified using Brilliant III SYBR green qPCR master mix (Agilent Technologies, Inc) on the QuantStudio 3 Real-Time PCR System (Applied Biosystems). All reactions were carried out in triplicates for each biological replicate. The *Ct* value target gene was normalized to GAPDH internal control to calculate ΔC*t* values. The difference in transcript levels between control (or scrambled) and treated (or knockdown) samples were determined by calculating fold change with 2^− ΔΔC*t*^ method as previously described [21].

### Immunoblotting

Human TNBC cells were exposed to base media control, tnLCM or tbLCM (SUM159) or vehicle control, recombinant mouse or human FGF2 (SUM159 and MDA-MB-231) for 72 h. Total protein was isolated using 1X RIPA lysis buffer. Lowry assays were performed to quantify total protein. Approximately 35 µg of protein per sample was used to assess expression of DACH1 using an anti-DACH1 antibody (1:1000, Clone 3B6D2, Cat# 60082-1 Proteintech, Rosemont USA) for 2 h at room temperature in 5% skimmed milk. β-actin (1:10,000) was used as a loading control (anti-human ACTB, Cat# A2066, Millipore Sigma, Darmstadt, Germany). Anti-human primary antibodies against stem cell markers such as SOX2 (1:1000, Clone 9-9-3, Cat# ab79351, Abcam, Boston, MA, USA), SOX9 (1:1000, Clone D8G8H, Cat# 82,630, Cell Signaling, Danvers, USA), OCT4 (1:1000, Clone C30A3, Cat# 2840, Cell Signaling, Danvers, MA, USA) and NANOG (1:1000, Clone D73G4, Cat# 4903, Cell Signaling, Danvers, MA, USA) were diluted in 5% skimmed milk and incubated overnight. Goat anti-rabbit and goat anti-mouse secondary antibody conjugated to horseradish peroxidase was used at a concentration of 1:5000 (Calbiochem, Billerica, MA, USA).

### TCGA analysis

Analysis of clinical breast cancer patient data from The Cancer Genome Atlas (TCGA) was performed using the ULCAN (http://ualcan.path.uab.edu/index.html) interactive web resource [22]. The TCGA breast invasive carcinoma data set was used to examine the expression of *DACH1* in breast cancer patient samples. Overall survival and progression free survival analysis was performed using Kaplan-Meier Plotter (https://kmplot.com/analysis/) interactive web resource [23].

### Statistical analysis

All in vitro experiments were performed in triplicate (*n* = 3) unless otherwise stated. Ex vivo experiments were carried out using multiple mice (PuMA: 3 mice/group/cell line; LCM: 4 mice/group). Statistical analyses were carried out using GraphPad Prism 6.0 (San Diego, CA, USA). Data are presented as the mean ± standard error of the mean (SEM). A two-way analysis of variance (ANOVA) was used to compare multiple means across different groups. In all case, p values of ≤ 0.05 were considered to be statistically significant.

## Results

### The lung microenvironment supports colonization and growth of breast cancer cells with an ALDH^hi^CD44^+^ stem-like phenotype

**Fig. 1:**
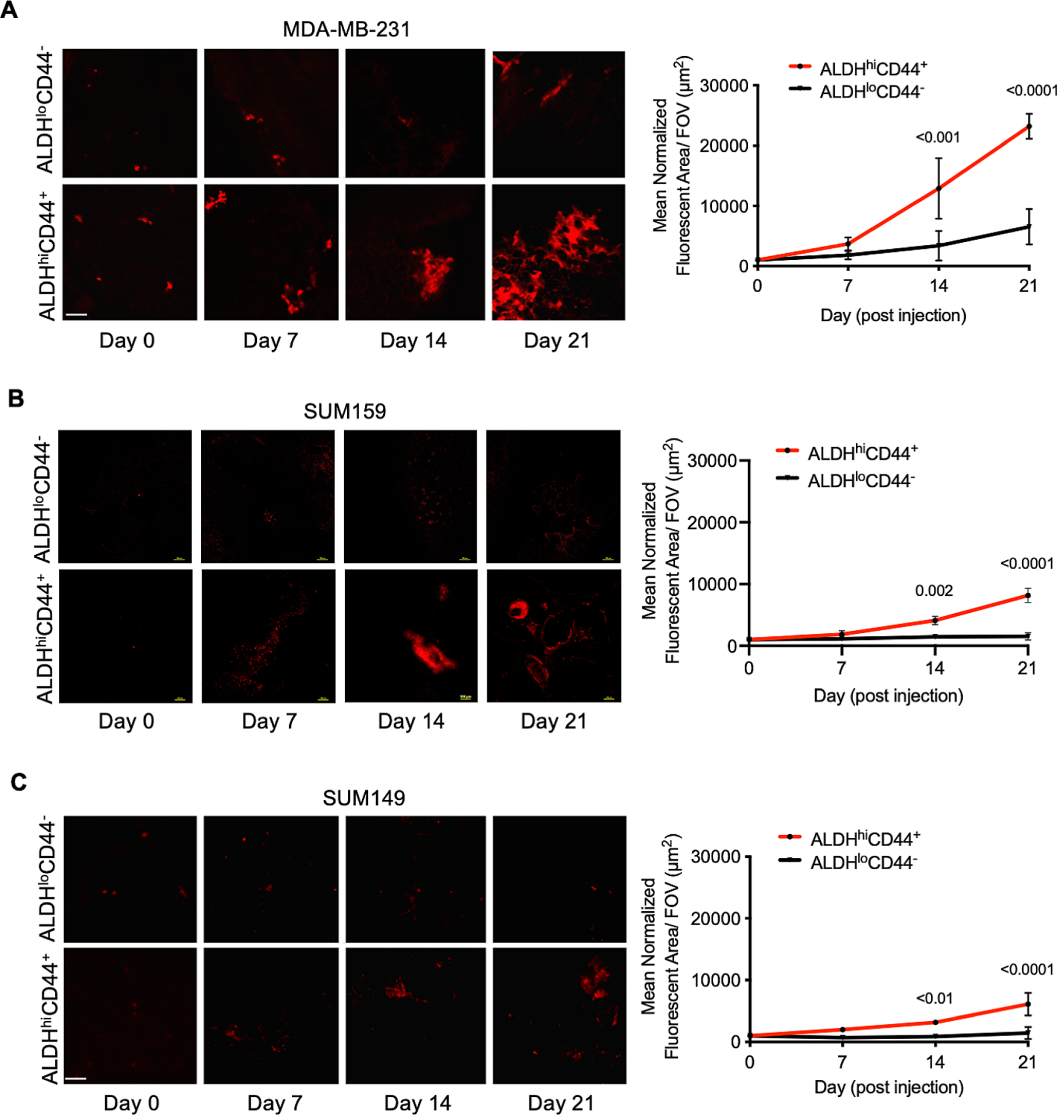
The lung microenvironment supports colonization and growth of breast cancer cells with an ALDH^hi^CD44^+^ stem-like phenotype: Breast cancer stem cells (ALDH^hi^CD44^+^) and non-breast cancer stem cells (ALDH^lo^CD44^−^) were isolated by FACS from MDA-MB-231, SUM159 and SUM149 breast cancer cells and delivered into lung to perform the ex vivo PuMA. Representative images show growth pattern of the subset of (**A**) MDA-MB-231, (**B**) SUM159 and (**C**) SUM149 breast cancer cells for each time point. Scale bar represents 100 μm. Progression of single cells to multicellular colonies by 21 was observed in breast cancer stem cell compartment. A mean normalized fluorescent area (µm^2^) per FOV was measured and averaged for each time point. Error bar represented as ± SD. Scale = 100 μm

**Fig. 2:**
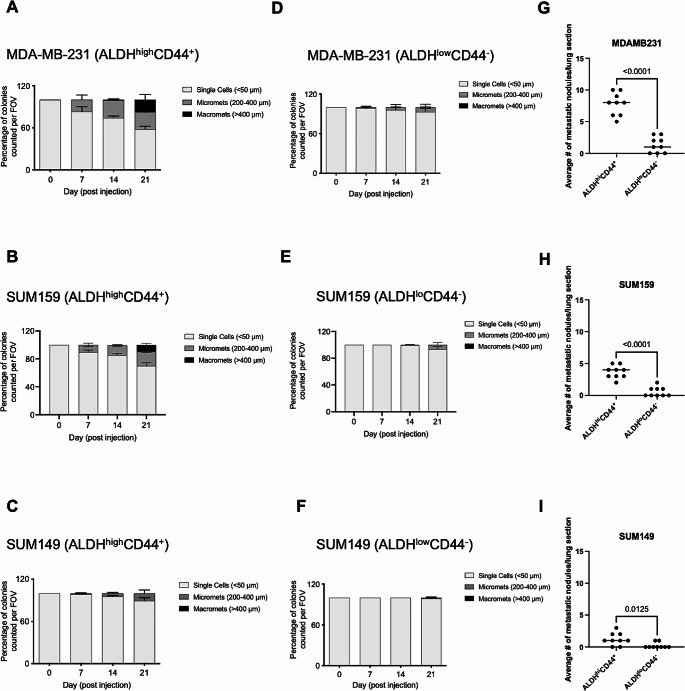
*Stem-like ALDH*^*hi*^*CD44*^*+*^*cells display metastatic progression from single cell to macrometastases in the lung*: Multicellular colonies generated by stem-like ALDH^hi^CD44^+^ and non-stem like ALDH^lo^CD44^−^ breast cancer cells were classified in diameters as single cells (50 μm), micrometastases (100–400 μm) or macrometastases (> 400 μm). Five random images were analyzed per lung section (*n* = 3 lung sections) for each time point. Only stem-like ALDH^hi^CD44^+^ cells from (**A**) MDA-MB-231 and (**B**) SUM159 breast cancer line progressed form single cells on day 0 to micrometastases by day 14 and macrometastases by day 21 as compared to non-stem like ALDH^lo^CD44^−^ cells. (**C**) ALDH^hi^CD44^+^ cells from SUM149 progressed from single cells to micrometastases by day 21. Majority of non-stem like breast cancer cells from all the breast cancer cell line remained as single cells throughout the assay. (**G**–**I**) Average number of metastatic nodules generated from stem-like ALDH^hi^CD44^+^ cells and non-stem like ALDH^lo^CD44^−^ breast cancer cells were assessed on day 21

We have previously observed that stem-like ALDH^hi^CD44^+^ breast cancer cells have a propensity to metastasize to the lung in vivo [15], and that the interaction between lung-derived soluble factors and CD44^+^ breast cancer cells supports metastatic behaviour [16]. Based on these findings, we hypothesized that the lung microenvironment may preferentially support the growth and colonization of ALDH^hi^CD44^+^ breast cancer cells relative to ALDH^lo^CD44^−^ cells. In order to investigate this further, we used an ex vivo pulmonary metastasis assay (PuMA) model [18, 19] to deliver FACS-isolated subsets of ALDH^hi^CD44^+^ and ALDH^lo^CD44^−^ breast cancer cells to the lung and to track the early steps of lung colonization and growth over 21 days of ex vivo culture. Although we observed that whole populations of different human breast cancer cell lines display varying degrees of growth in the PuMA (*Supplemental Figure **S2**B-D*), in all TNBC cell models we observed that ALDH^hi^CD44^+^ cells displayed enhanced colonization and growth in the lung microenvironment relative to ALDH^lo^CD44^−^ cells (*p*≤0.05; Fig. 1A-C). To further quantify the differences in progression within the lung between stem-like ALDH^hi^CD44^+^ and non-stem-like ALDH^lo^CD44^−^ breast cancer cells, we measured and classified the size of multicellular colonies present in the PuMA according to diameter as single cells (≤ 50 μm), micrometastases (100–400 μm) or macrometastases (> 400 μm). Stem-like ALDH^hi^CD44^+^ cells from the highly aggressive SUM159 and MDA-MB-231 cell lines progressed from single cells at day 0 to micrometastases by days 7 and 14, and to macrometastases by day 21 (Fig. 2A-B). In the weakly metastatic SUM149 cell line, stem-like ALDH^hi^CD44^+^ progressed from single cells at day 0 to micrometastases by day 21 (Fig. 2C). In contrast, the majority of non-stem-like ALDH^lo^CD44^−^ cells remained as single cells from day 0 throughout the duration of the assay to day 21 (Fig. 2D-F). Additionally, on day 21 we observed an increase in the number of metastatic nodules formed by stem-like ALDH^hi^CD44^+^ cells as compared to non-stem-like ALDH^lo^CD44^−^ cells (Fig. 2G-I). Taken together, these data suggest that the lung microenvironment provides a supportive niche for metastatic colonization and growth of ALDH^hi^CD44^+^ breast cancer cells.

### Soluble factors from lungs primed by the presence of a TNBC primary tumor can modify expression of stem cell-related genes in breast cancer cells

**Fig. 3:**
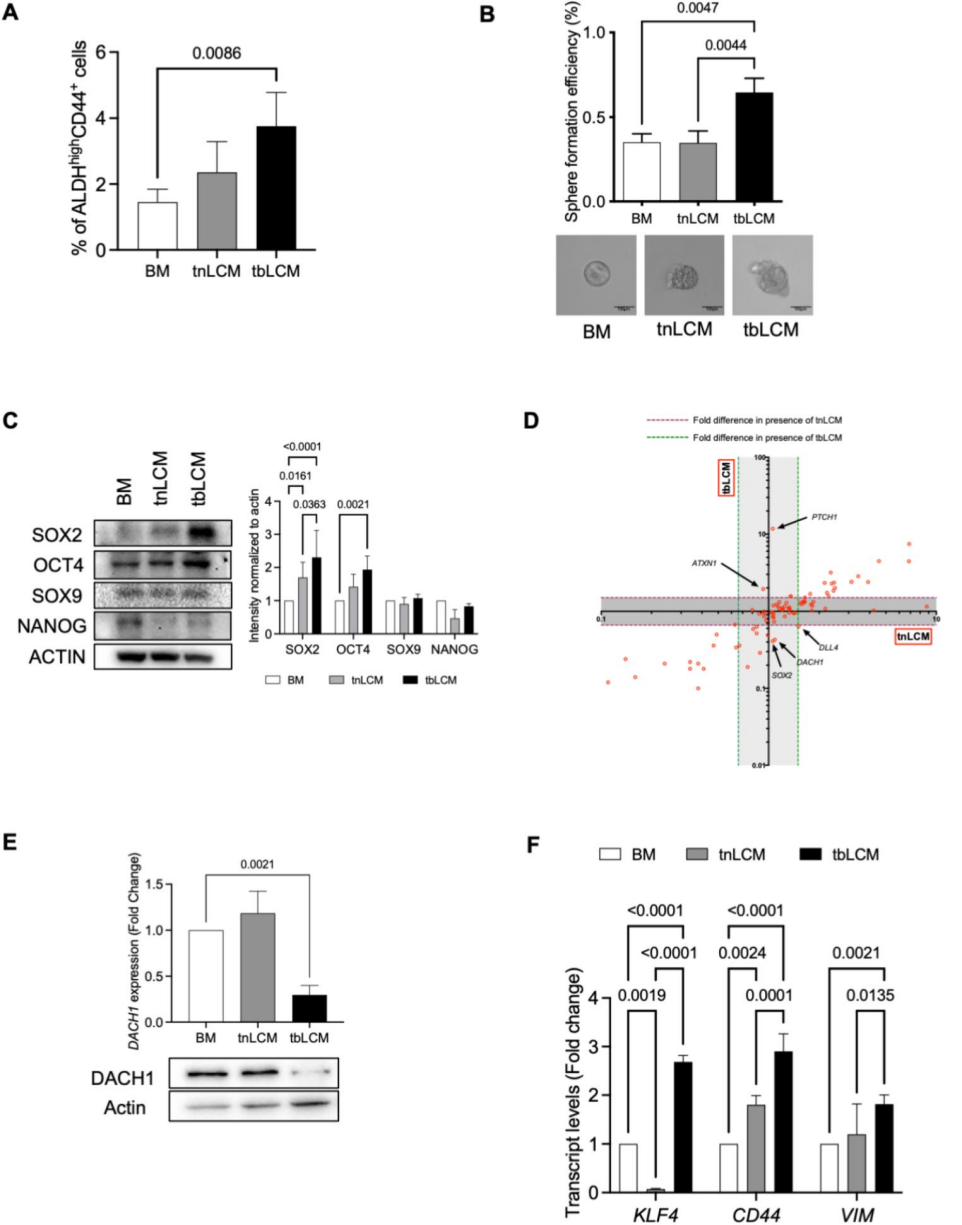
*Lung-derived soluble factors influence stemness/plasticity via down regulation of DACH1 expression*: SUM159 breast cancer cells were exposed to basal media (BM) control, or lung conditioned media (LCM) from tumor naïve (tnLCM) and SUM159 tumor bearing (tbLCM) mice for 72 h and assessed by flow cytometry (**A**) for differences in ALDH^hi^CD44^+^ phenotype and mammosphere assay (**B**) for sphere forming ability. (**C**) Expression of stem-cell related genes including SOX2, SOX9, OCT4 and NANOG was assessed in SUM159 breast cancer cells exposed to BM control, tnLCM and tbLCM (*n* = 3). (**D**)Total RNA was isolated from SUM159 breast cancer cells exposed to BM or tnLCM and tbLCM and human cancer stem cell RT2 PCR array was performed to examine cell intrinsic changes. Arrow shows genes differentially regulated in tbLCM treated breast cancer cells as compared to tnLCM. Both are normalized to BM control (*n* = 3). (**E**) SUM159 breast cancer cells exposed to tbLCM showed decrease in expression of DACH1 protein levels as compared to base media BM or tnLCM. (**F**) SUM159 breast cancer cells exposed to tbLCM increased transcript levels of DACH1 target genes as compared to BM or tnLCM. Data are presented as the mean ± SD (*n* = 3)

We recently reported that stromal and soluble components of the lung microenvironment can be modified by the presence of a primary TNBC tumor [17], and thus we hypothesized that this might also affect breast cancer stemness/plasticity. To assess this, we injected SUM159 human TNBC cells into the mammary fat pad of nude mice to generate primary tumors, with age-matched tumor-naïve mice serving as healthy controls. Lungs were harvested 4 weeks post injection and lung-conditioned media (LCM) was generated as described previously [16, 17]. We exposed breast cancer cells to LCM from tumor-naïve mice (tnLCM) or from mice bearing TNBC primary tumors (tbLCM) and assessed for changes in ALDH^hi^CD44^+^ phenotype. We observed that SUM159 TNBC cells exposed to tbLCM demonstrated a ~ 1.6-fold increase in acquisition of a stem-like ALDH^hi^CD44^+^ phenotype relative to tnLCM and ~ 2.6-fold increase relative to BM control (Fig. 3A; *p* = 0.0086). In keeping with this, exposure to tbLCM also resulted in a significant increase in the mammosphere-forming ability of SUM159 cells (Fig. 3B; *p* = 0.0047). We next investigated changes in expression of specific stem cell markers by immunoblotting, including SOX2, SOX9, OCT4 and NANOG (Fig. 3C). We observed that SOX2 and OCT4 expression was significantly increased in tbLCM-treated breast cancer cells as compared to tnLCM or BM treated cells, while expression of SOX9 and NANOG was not different between treatment conditions. For a broader assessment of whether soluble lung-derived factors could modify cell-intrinsic factors related to stemness, SUM159 TNBC cells were exposed to BM, tnLCM and tbLCM for 72 h and changes in gene expression were assessed using a Human Cancer Stem Cell RT2 Profiler PCR® array. We observed that several genes including *PTCH1*, *ATXN1*, *DLL4*, *SOX2* and *DACH1* were differentially regulated in tbLCM-treated breast cancer cells as compared to those treated with tnLCM or BM control (Fig. 3D). Notably, Dach1 (Dachshund homolog 1) is transcription factor and a putative tumor suppressor that suppresses breast cancer tumor growth, spread and stemness [24–26]. Further RT-qPCR and immunoblotting validation revealed that treatment with tbLCM significantly reduced *DACH1* protein expression (Fig. 3E; *p* = 0.0021). Furthermore, treatment with tbLCM increased stemness-related transcriptional targets of Dach1, including *KLF4*, *CD44* and *Vimentin* [26] (Fig. 3F). These data suggest that soluble lung-derived factors can influence plasticity/stemness, resulting in an enhanced stem-like phenotype and gene expression profile in TN breast cancer cells.

### Low expression of DACH1 is associated with poor progression free survival, overall survival and enhanced stemness/plasticity.

**Fig. 4:**
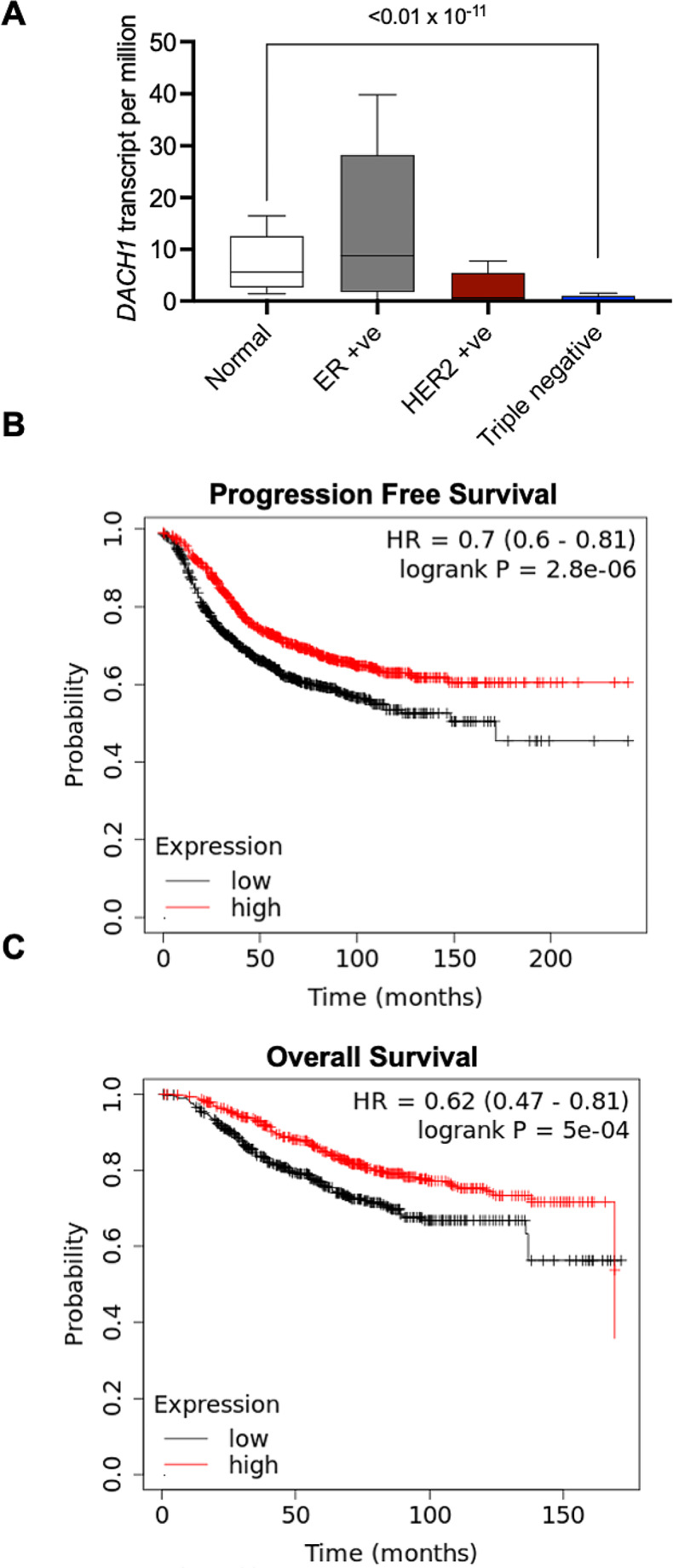
*DACH1 expression levels dictate clinical outcomes*: (**A**) TCGA analysis show significant reduction in *DACH1* transcript levels in triple negative breast cancer subtype as compared to normal breast tissue samples. Kaplain Meier (KM) plot was generated using kaplain-meier plotter web-based tool. In this analysis, median progression free survival (PFS) and overall survival (OS) was calculated using breast cancer data from all subtypes (PFS, *n* = 2032; OS, *n* = 943). A cut off of 254 for PFS and 375 for OS was applied. Survival is shown in months. KM plot shows significant decrease in (**B**) progression free survival and (**C**) overall survival in breast cancer patients with lower DACH1 expression

**Fig. 5:**
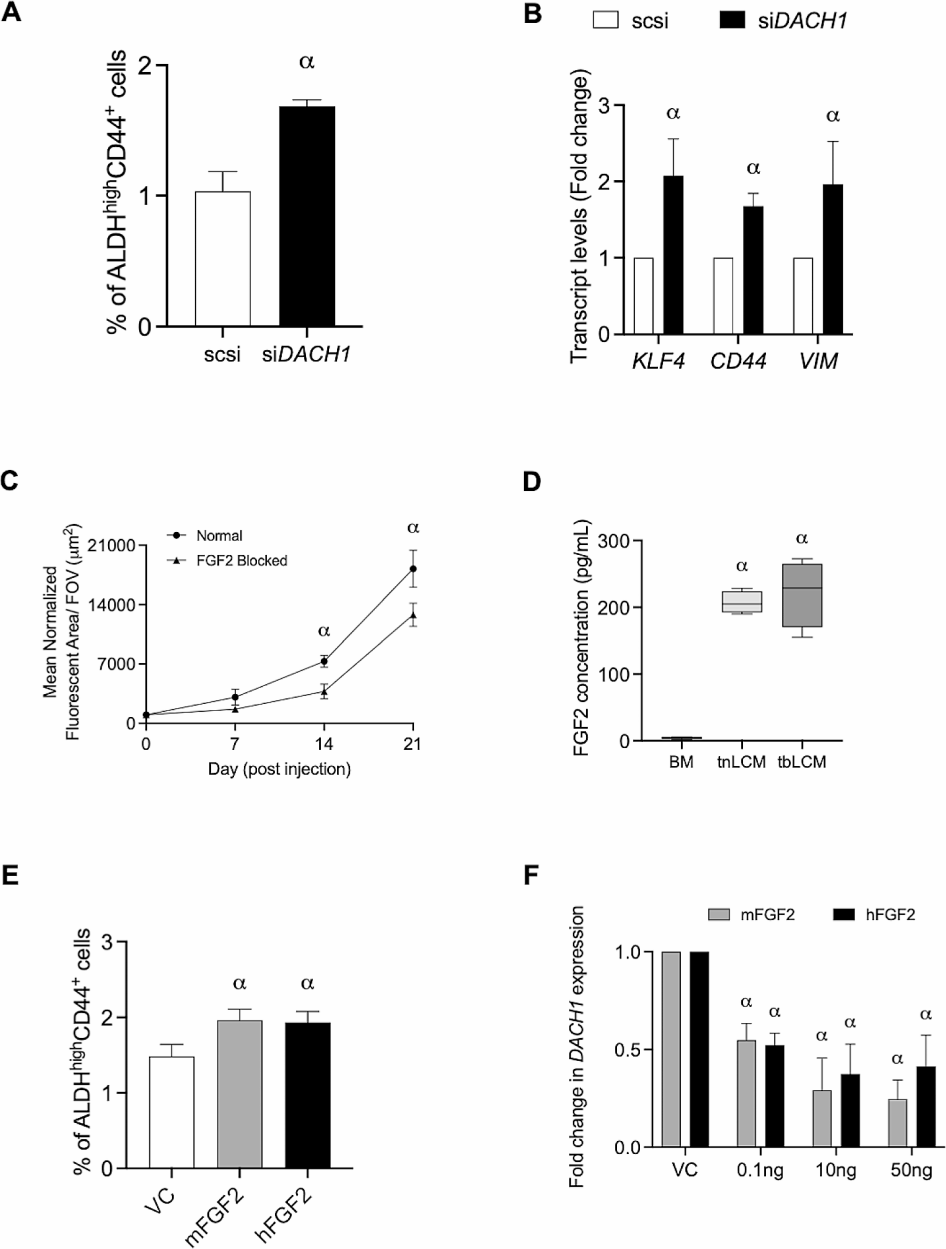
*FGF2-DACH1 signaling axis enhances breast cancer metastatic colonization and stemness/plasticity:* (**A**) SUM159 cells were transfected with either non-targeting (scsi) or *DACH1* targeting (si*DACH1*) siRNA. Loss of DACH1 expression in SUM159 breast cancer cells increases acquisition of stem-like ALDH^hi^CD44^+^ phenotype as compared to scsi control (*p* ≤ 0.05). Data are presented as the mean ± SD (*n* = 3). (**B**) Loss of *DACH1* expression in SUM159 breast cancer cells resulted in increased transcript levels of DACH1 target genes. 𝛼 = statistically significant increase in gene expression as compared to scsi control (*p* ≤ 0.05). (**C**) The role of FGF2 on colonization and growth of breast cancer cells in the ex vivo PuMA was measured by delivering MDA-MB-231 breast cancer cells to lungs infused with a neutralizing FGF2 antibody. Lung colonization of breast cancer cells was significantly reduced in the presence of neutralizing FGF2 antibody (*p* ≤ 0.05) (**D**) Levels of soluble FGF2 in lung conditioned media (LCM) generated from tumor naïve (tnLCM), SUM159 tumor bearing (tbLCM) and base media (BM) control determined by ELISA. Data are presented as mean ± SD, 𝛼 = significantly different than control (*p* ≤ 0.05). (**E**) SUM159 was exposed to 10ng of recombinant mouse (mFGF2) and human (hFGF2) for 72 h and stemness was assessed by flow cytometry. Data are presented as mean ± SD, 𝛼 = significantly different than control (*p* ≤ 0.05; *n* = 3). (**F**) Treatment with both recombinant mFGF2 and hFGF2 resulted in significant decrease in DACH1 transcript in SUM159 cells (*p* ≤ 0.05). Data are presented as the mean ± SD (*n* = 3)

Analysis of breast cancer patient data through TCGA revealed that *DACH1* expression is significantly downregulated in TNBC as compared to normal breast tissue (Fig. 4A; *p*≤0.01 × 10^− 11^). In addition, low expression of *DACH1* is associated with poor progression free survival and overall survival (Fig. 4B, C; *p*≤0.05 × 10^− 2^). To investigate if Dach1 also has a functional role in influencing the ALDH^hi^CD44^+^ phenotype, siRNA was used to knockdown expression of *DACH1* in SUM159 and MDA-MB-231 TNBC cells (*Supplemental Figure **S3**A, B*; *p*≤0.05). Inhibition of *DACH1* significantly increased acquisition of a stem-like ALDH^hi^CD44^+^ phenotype (Fig. 5A, *Supplemental Figure **S3**C; p*≤0.05). We also observed that loss of *DACH1* in TNBC cells resulted in increased expression of its targets such as *KLF4*, *CD44* and *Vimentin* (Fig. 5B), in a manner similar to that previously observed when TNBC cells were exposed to tbLCM (Fig. 3E). Taken together, these results further support the concept that soluble factors in the primed lung microenvironment influence stemness/plasticity in TN breast cancer cells through suppression of *DACH1* expression.

### The FGF2-DACH1 signaling axis enhances breast cancer metastatic colonization and stemness/plasticity

Finally, to further understand the role of lung microenvironment in supporting growth and colonization of breast cancer cells, we set out to identify potential lung-derived soluble factors associated with growth and colonization of breast cancer cells. Previous studies have shown that FGF2 expression was transcriptionally suppressed by Dach1 in glioma cells [27] and on the contrary, FGF2 was shown to activate *DACH1* during skeletal development in mouse [28]. This led us to investigate the potential role of FGF2-DACH1 signaling axis in the lung microenvironment. Additionally, we had previously identified several lung-derived soluble proteins associated with cell adhesion, stemness/plasticity, migration, and neoplasia including fibroblast growth factor 2 (FGF2) [16]. FGF2 has been associated with regulation and maintenance of normal stem cells [29]. This led us to investigate its role in metastatic growth and lung colonization of breast cancer cells. To test this, we allowed MDA-MB-231 TNBC cells to grow in the ex vivo PuMA either in the presence or absence of a neutralizing FGF2 antibody. We observed that blocking of FGF2 protein by the neutralizing antibody resulted in significant reduction in growth and colonization of breast cancer cells at days 14 and 21 relative to control (Fig. 5C; *p*≤0.05). This suggests that lung-derived soluble factor FGF2 may be required for growth and colonization of breast cancer cells. We also observed that LCM generated from either tumor-naïve mice (tnLCM) or from mice bearing TN primary tumors (tbLCM) had a significantly increased concentration of FGF2 compared to control media (Fig. 5D; *p* ≤ 0.05). Next, we were interested in investigating whether the presence of FGF2 could lead to dynamic changes in the ALDH^hi^CD44^+^ phenotype in breast cancer cells. Breast cancer cells were treated with recombinant mouse (mFGF2) or human (hFGF2) FGF2 protein for 72 h and assessed for changes in stem-like ALDH^hi^CD44^+^ phenotype via flow cytometry. Compared to vehicle control, mFGF2 and hFGF2 treated breast cancer cells displayed and enhanced stem-like ALDH^hi^CD44^+^ phenotype (Fig. 5E *and Supplementary Fig. 3D; p*≤0.05). To test this, we treated breast cancer cells with recombinant FGF2 protein and observed a dose dependent decrease in *DACH1* transcript levels as compared to vehicle control (Fig. 5F; *p*≤0.05). We also observed that 3 h of 100ng of recombinant FGF2 was sufficient to significantly decrease Dach1 protein levels (*Supplementary Fig. 4A*). Finally, we assessed acquisition of stem-like ALDH^hi^CD44^+^ phenotype in breast cancer cells treated with BM, tnLCM or tbLCM in the presence or absence of a neutralizing monoclonal FGF2 antibody. We observed a marginal increase in Dach1 protein levels in tbLCM + anti-FGF2 treated cells as compared to tbLCM treatment alone (*Supplementary Fig. 4B&C*). Additionally, we observed a minor decrease (~ 10%) in acquisition of stem-like ALDH^hi^CD44^+^ phenotype in tbLCM + anti-FGF2 treated cells as compared to tbLCM treatment alone (*Supplementary Fig. 4D*). Taken together, this data suggested that activation of Fgf2 signaling may decreases *DACH1* expression to promote metastatic colonization and stemness/plasticity in breast cancer cells.

## Discussion

Metastasis is a major cause of mortality and morbidity in breast cancer patients [1, 2]. The lung is one of the most common and deadliest sites of metastasis, especially for the most aggressive TNBC subtype [12, 30]. Despite advancements in current multimodality therapy options such as chemotherapy and radiotherapy, the median survival of breast cancer patients with lung metastases is approximately 11 months [12]. Several studies have shown that orchestrated interactions between disseminated cancer cell and metastatic microenvironment play a critical role in survival of cancer cells and formation of metastatic lesions [31–33]. Therefore, investigating the relationship between disseminated cancer cells and metastatic microenvironment will help in designing the most effective therapy options to treat or prevent lung metastasis. In this study we demonstrate that lung-derived soluble factors secreted in the presence of primary TNBC tumor play a critical role in influencing stemness/plasticity and metastatic behavior of TNBC cells.

Metastasis is a highly complex and an inefficient process as only a small percentage of disseminated cancer cells survive and grow at the secondary site following extravasation [7, 8]. Survival and growth of disseminated tumor cells mainly depends on cell-intrinsic (stemness/plasticity) and cell-extrinsic (favourable microenvironment at the secondary site) factors and the interaction between the two [34]. The preferential metastasis of TNBC to the lung suggests that lung provides a favourable nice for survival of these breast cancer cells. Even though breast cancer cells are successful in surviving and growing in the primary site, they often fail to grow and colonize distant organs [35]. This suggests that breast cancer cells with specific characteristics may have a better chance of survival and growth at metastatic sites. Our previous work has demonstrated that stem-like breast cancer cells, defined as cells with high ALDH activity and CD44 expression, displayed enhanced metastatic potential [15] and preferential metastasis to the lung [16].

In this current study, we observed that the lung microenvironment favoured growth and colonization of breast cancer cells with stem-like ALDH^hi^CD44^+^ characteristics as compared to non stem-like ALDH^lo^CD44^−^ breast cancer cells. We observed that, even though the whole cell population of TN breast cancer cells modestly colonized the lung, the ALDH^hi^CD44^+^ enriched subset of most aggressive SUM159 and MDA-MB-231 TNBC cells, displaying enhanced lung colonization and progression from single cell state to macrometastases by 21 days in the ex vivo pulmonary metastasis assay. The ability of stem-like cells to progress from single cell to macromateastases suggests that stemness/plasticity may be a key component for survival and metastatic colonization especially in the lung. Previously *Pein et al.* showed that disseminated breast cancer cells with stem-like feature can induce pre-metastatic changes in lung fibroblasts to favor their survival and colonization [36]. In this study it is worth noting that in the ex vivo PuMA model, growth and colonization of stem-like ALDH^hi^CD44^+^ breast cancer occurred in the absence of pre-metastatic niche. This suggests that stem-like ALDH^hi^CD44^+^ cells may have possibly remodelled the lung microenvironment for survival and colonization after arrival in the lung. In addition, we also observed that even though the non stem-like breast cancer cells survived, they remain as single cells through in the ex vivo PuMA model. This suggests that a pre-metastatic niche may be required for colonization of breast cancer cells that lacking stem-like characteristics. It can be speculated that these non stem like single cells may remain dormant and upon acquisition of favourable conditions they could acquire stem-like phenotype and progress to macrometastasis. Several studies have demonstrated the stemness/plasticity often hinders effective therapy response through development of resistance to therapy [37, 38]. Therefore, elucidating the mechanism that confers stemness/plasticity would be beneficial in better treatment outcomes in metastatic patients. Taken together our results suggests that stem-like cell intrinsic factors that confer survival ability in an unfavourable microenvironment is very critical for successful metastasis. However, the mere presence of breast cancer cells with stem-like features does not always contribute to successful metastasis, suggesting that in addition to cell-intrinsic high ALDH activity and expression of CD44 in breast cancer cells, a supportive microenvironment is critical for successful metastatic process. growth and metastatic colonization breast cancer cells.

Stephen Paget’s “Seed and Soil” hypothesis proposes that a favourable microenvironment (soil) is critical for survival and growth of breast cancer cells (seed) at distant sites [10]. Interaction between disseminated tumor cells and the microenvironment at distant site is critical for metastasis [34]. Our previous work showed that presence of a primary TNBC breast tumor preferentially ‘primes’ the lung microenvironment thereby changes the secretion profiles of lung-derived soluble factors [17]. The primed lung microenvironment might be ideal in inducing stemness/plasticity in non stem-like breast cancer cells. In this study, we found that breast cancer cells exposed to LCM from TNBC-tumor bearing mice significantly increased acquisition of a stem-like phenotype and significantly decreased expression of a potential tumor suppressor, Dach1. Dach1 is a transcription factor involved in regulating different function during development [39]. In addition, it is known to suppress tumor cell growth [40–42], migration/invasion [24] stemness [26, 43] and metastasis [24]. We observed that decreased expression of *DACH1* in TNBC breast cancer patients was associated with poor overall survival and progression free survival and that inhibition of *DACH1* significantly increased acquisition of a stem-like ALDH^hi^CD44^+^ phenotype in TNBC cells. Dach1 is known to negatively regulate stemness and inhibits expression of stem cell markers *KLF4* and *CD44*, and epithelial to mesenchymal transition marker *Vimentin* [26]. We also found that tbLCM exposure resulted in increase in classical stem cell markers including SOX2 and OCT4 protein levels as compared to BM or tnLCM. Furthermore, exposure of tbLCM or loss of function of *DACH1* in breast cancer cells significantly increased expression of *KLF4*, *CD44* and *Vimentin*, indicating that one or more secreted factors in the LCM derived from TNBC tumor-bearing mice influences stemness/plasticity in breast cancer cells.

To this end, we found that previously identified [16] lung-derived soluble factor FGF2 was important for metastatic colonization of TN breast cancer cells. FGF2 is known to play a key role in different cellular processes such as development [29], maintenance of normal stem cells [44, 45], cellular proliferation [46, 47], and angiogenesis [48]. It is also known to regulate cancer stem cell function [49, 50]. In this study we found that TNBC cells exposed to recombinant FGF2 resulted in significant increase in acquisition of stem-like ALDH^hi^CD44^+^ phenotype however the increase was moderate as compared to that observed in presence of the tbLCM. Furthermore, when we quantified the levels of FGF2 in tbLCM, to our surprise, we did not observe any significant difference in the levels of soluble FGF2 in lung conditioned media from tumor bearing (tbLCM) mice as compared to tumor naïve mice. Additionally, blocking FGF2 signaling in breast cancer cells in the presence of BM, tnLCM or tbLCM had minimal influence on inhibiting acquisition of stem-like ALDH^hi^CD44^+^ phenotype. Previously we identified 16 lung-derived soluble factors unique to tbLCM as compared to tnLCM [17] and these factors together may compensate for the loss of FGF2 signaling. This suggested that TNBC cells in the lung may rely on other lung-derived soluble in addition to FGF2 in influencing their stemness/plasticity.

Activation of FGF2 signaling results in perturbation of multiple signaling pathways and genes related to different cellular processes [29]. FGF2 secreted by osteogenic cells in the bone microenvironment induces stemness/plasticity in ER^+^ breast cancer cells by suppressing ER expression [51]. Interestingly, DACH1 is also known to suppress ERα in breast cancer cells [52]. This suggests a possible link between FGF2 and DACH1 in regulating different cellular processes. Interestingly, during skeletal development FGF2 upregulated *DACH1* expression [28]. In preosteoblast cells, Dach1 played a key role in inhibiting FGF2 induced RANKL gene expression [53]. However, in glioma, Dach1 has been shown to transcriptionally suppress *FGF2* expression [27]. This suggests a time and context dependent role of FGF2-Dach1 signaling axis in regulating different cellular function. In our current study, we found that TNBC cells treated with recombinant FGF2 protein significantly downregulated DACH1 expression. In addition, we also observed increase in expression of *KLF4*, *CD44* and *Vimentin* expression which mimicked the loss of function of DACH1. Taken together these results suggested a link between FGF2 and Dach1 in influencing stemness/plasticity in breast cancer cells.

**Fig. 6:**
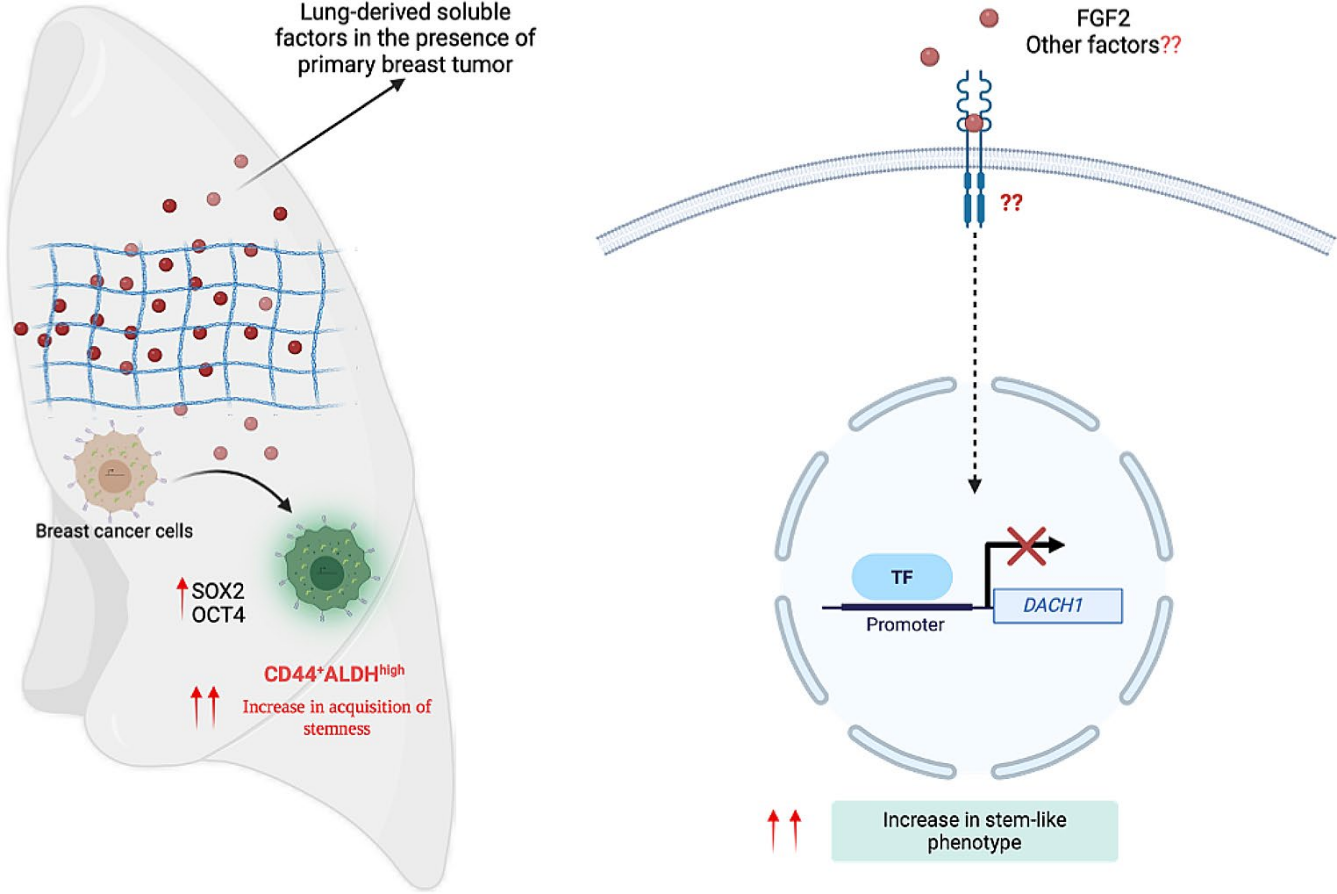
*Schematic showing the influence of lung-derived soluble factors on stemness/plasticity of breast cancer cell.* Lung-derived soluble factors, along with FGF2 secreted in the presence of primary breast tumor suppressed expression of a tumor suppressor DACH1 and enhanced acquisition of stemness/plasticity of breast cancer cells. *Image created using BioRender.com*

In conclusion, our study has demonstrated that the lung-derived soluble factors, particularly those secreted in the presence of primary TNBC tumor, play an important role in influencing stemness/plasticity and metastatic behaviour of breast cancer cells. Furthermore, we show that the FGF2-Dach1 signaling axis supports acquisition of stem-like ALDH^hi^CD44^+^ phenotype that is mostly favoured by the lung microenvironment for growth and metastatic colonization of the lung (Fig. 6I). We also showed that loss of *DACH1* inhibited metastatic outgrowth in the lung suggesting that FGF2 and other soluble factors secreted in the microenvironmental may play a critical role in regulating DACH1 expression and orchestrate metastatic behavior of breast cancer cells. This study highlights the complex interplay between FGF2, DACH1 and the lung microenvironment in influencing the stemness and metastatic behaviour of breast cancer cells. Future studies involving shRNA knockdown of DACH1 and FGF2 overexpression are required to further understand the role of FGF2-DACH1 signaling axis in the regulation of lung metastasis. This pre-clinical work lays the foundation for future evaluation of FGF2 as a potential novel therapeutic target for treatment or prevention of breast cancer metastasis to the lung.

## Electronic supplementary material

Below is the link to the electronic supplementary material.

Supplementary Material 1

Supplementary Material 2

Supplementary Material 3

Supplementary Material 4

Supplementary Material 5

Supplementary Material 6
