# Supplementary figures and images for: Lung-derived soluble factors support stemness/plasticity and metastatic behaviour of breast cancer cells via the FGF2-DACH1 axis
Source: Clin Exp Metastasis. 2024 Apr 6;41(5):717–31. doi: 10.1007/s10585-024-10284-4 (PMC11499378; doi:10.1007/s10585-024-10284-4)

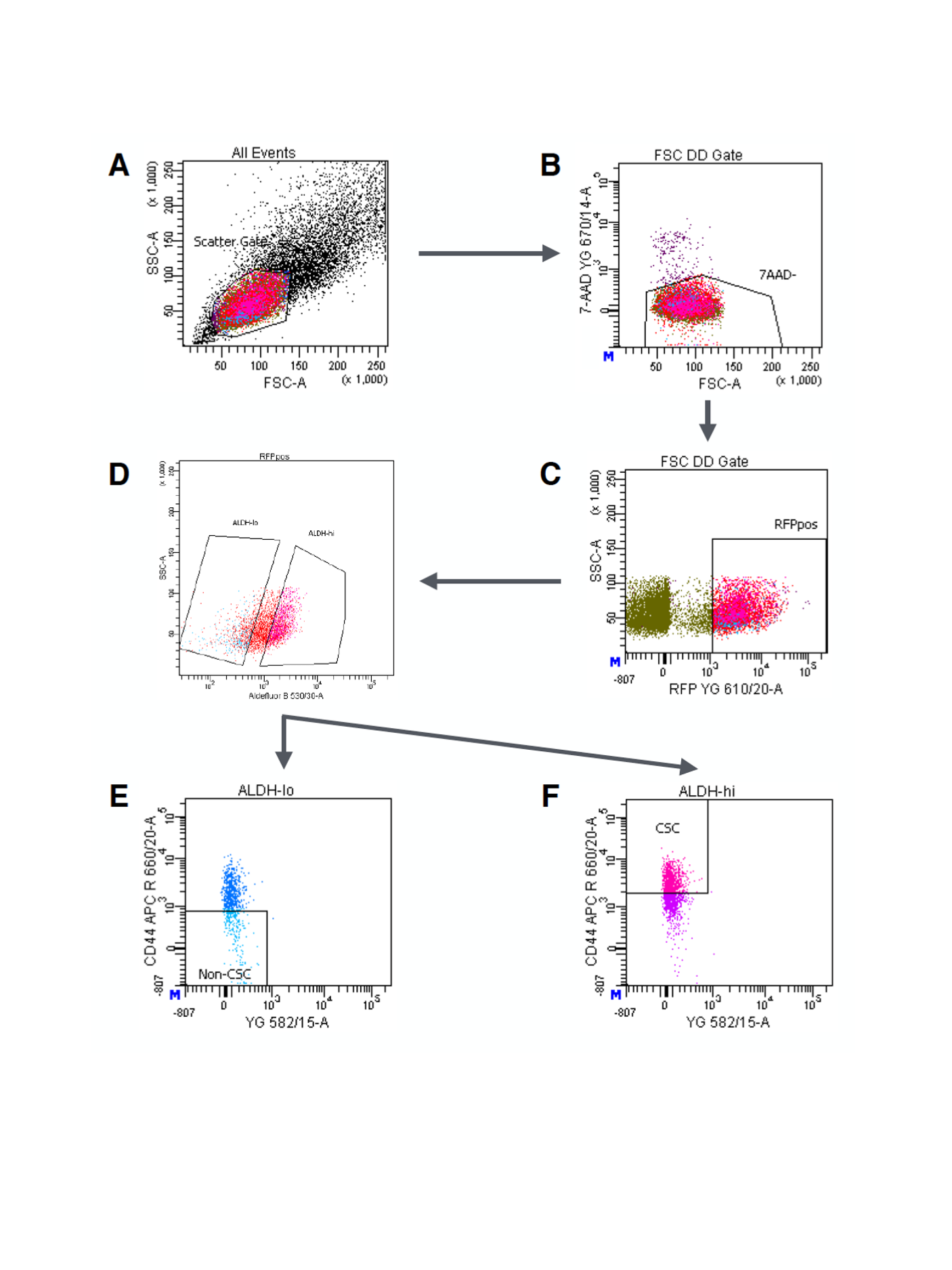

Supplement: Supplementary file 2 — Supplementary Material 2 [file 10585_2024_10284_MOESM2_ESM.tiff]

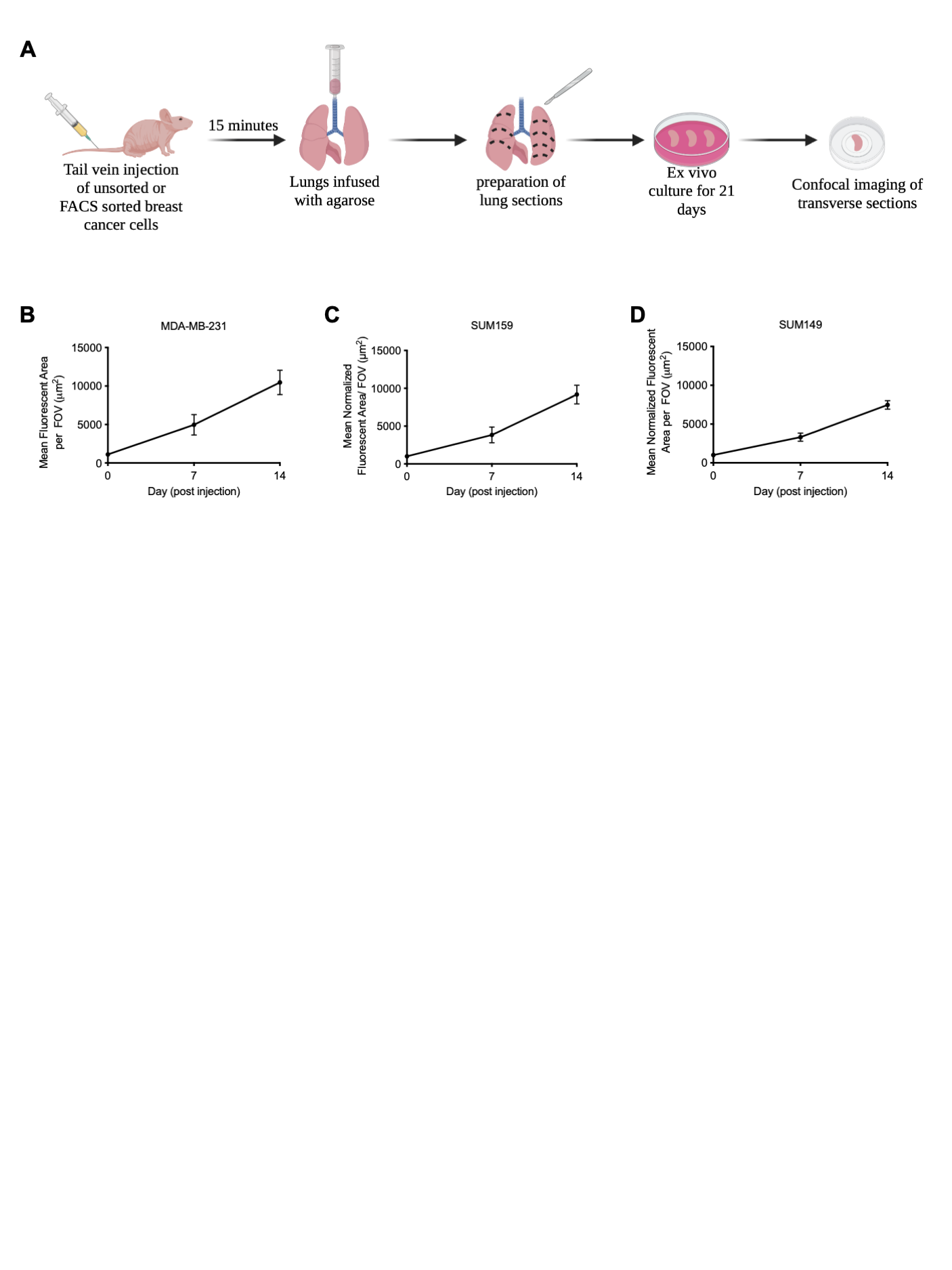

Supplement: Supplementary file 3 — Supplementary Material 3 [file 10585_2024_10284_MOESM3_ESM.tiff]

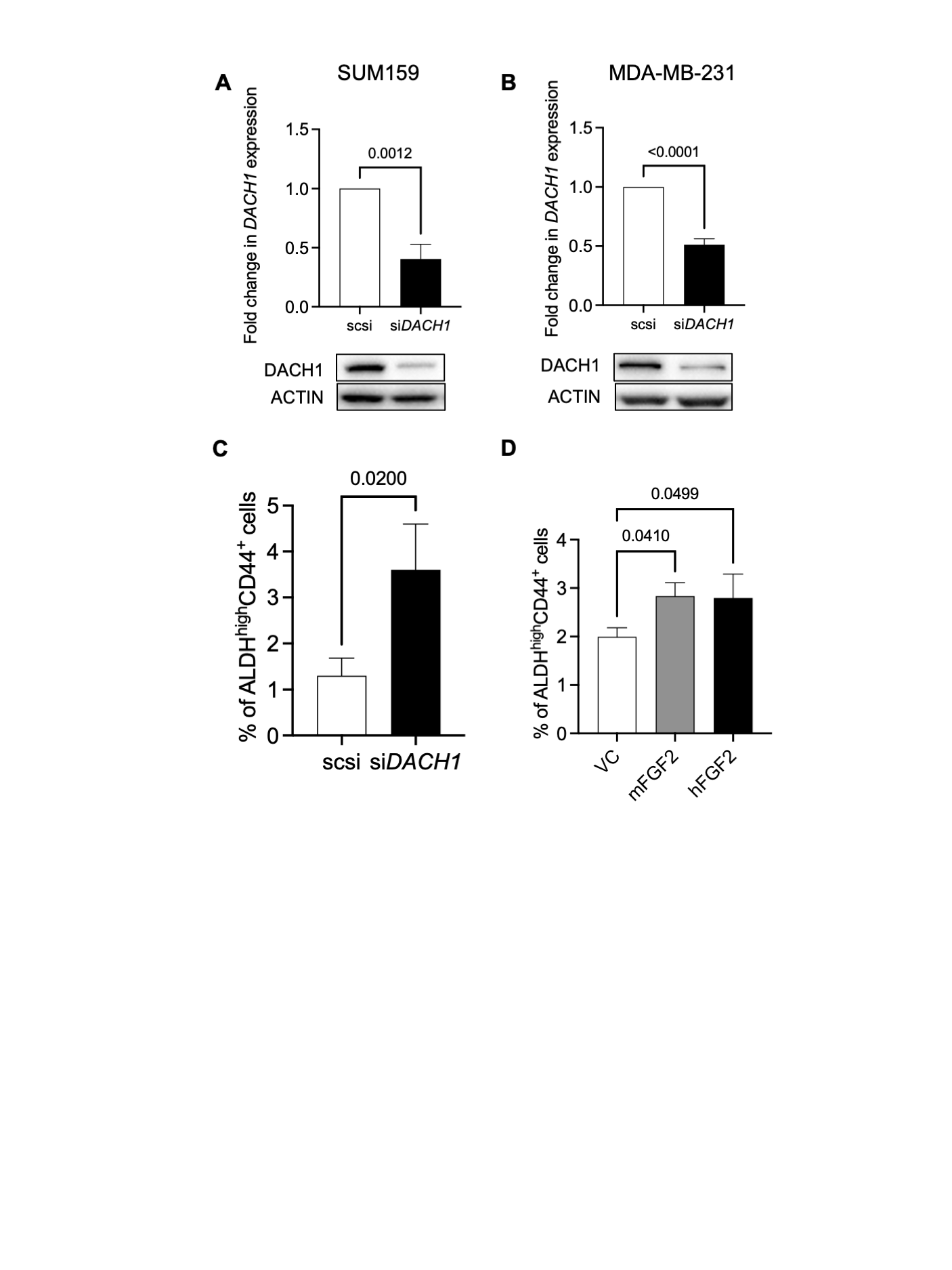

Supplement: Supplementary file 4 — Supplementary Material 4 [file 10585_2024_10284_MOESM4_ESM.tiff]

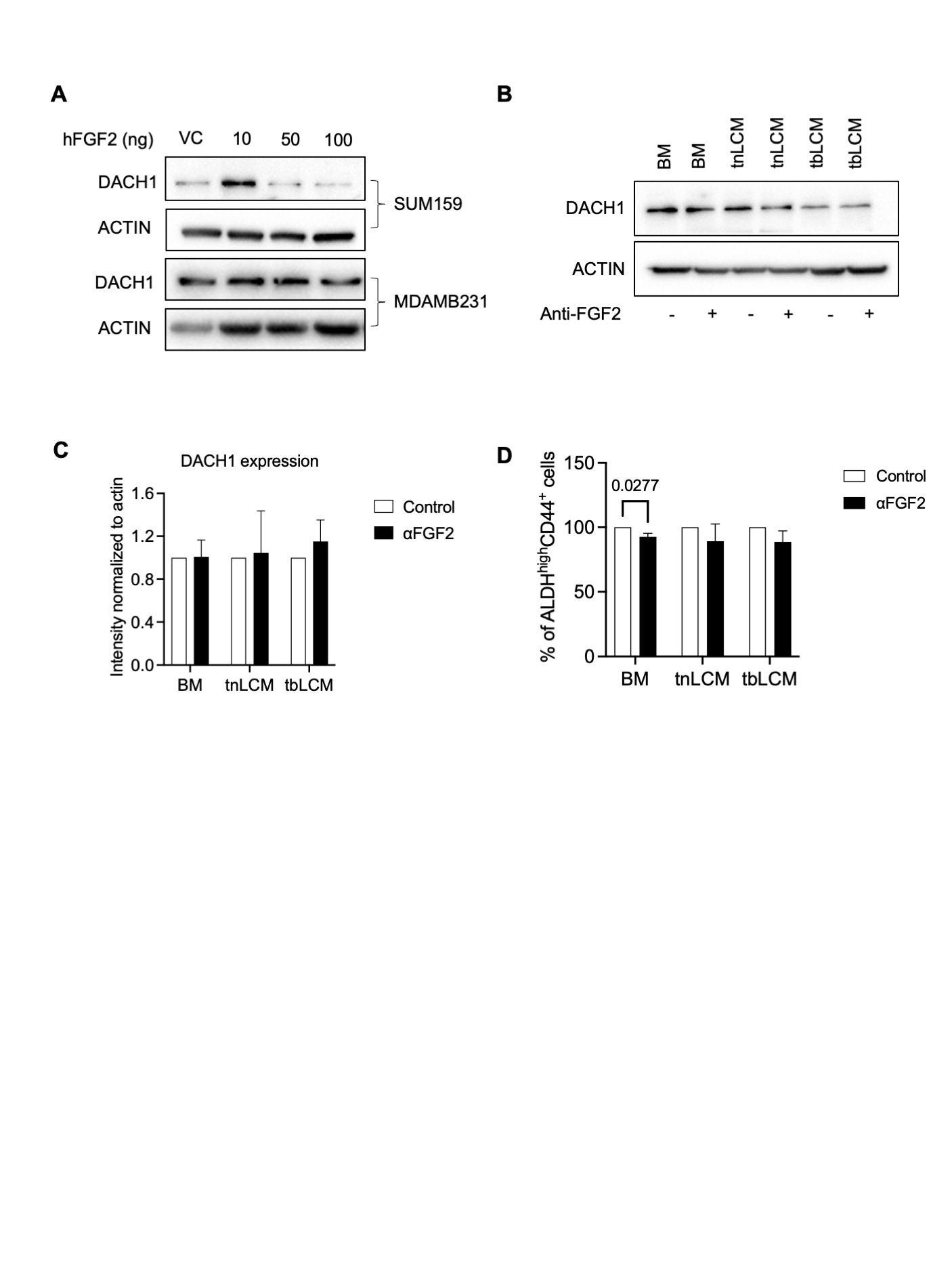

Supplement: Supplementary file 5 — Supplementary Material 5 [file 10585_2024_10284_MOESM5_ESM.tiff]
