# Supplementary material for: Lung-derived soluble factors support stemness/plasticity and metastatic behaviour of breast cancer cells via the FGF2-DACH1 axis
Source: Clin Exp Metastasis. 2024 Apr 6;41(5):717–31. doi: 10.1007/s10585-024-10284-4 (PMC11499378; doi:10.1007/s10585-024-10284-4)
Supplement: Supplementary file 6 — Supplementary Material 6 [file 10585_2024_10284_MOESM6_ESM.docx]

***Supplementary Fig. 1: Schematic representing strategy for isolation of stem-like ALDH^hi^CD44^+^ and non-stem-like ALDH^lo^CD44^−^ human triple negative breast cancer cells.*** Fluorescence activated cell sorting (FACS) was used to isolate both ALDH^hi^CD44^+^ and ALDH^lo^CD44^−^ cell populations labelled with 7-AAD, CD44-APC and the Aldefluor™ assay kit. Cell subsets were isolated using a four-colour protocol on a FACS ARIA I and subsequently used for the PuMA. (A) Cells were first selected based on expected light scatter, (B) viability based on 7-AAD exclusion, (C) and tdTomato positivity for MDA-MB-231 and mCherry positivity for SUM159 and SUM149 cells. (D) Cells were further divided into ALDH^hi^ and ALDH^lo^ populations. (E) Finally, cells were further selected based on a CD44^+^ phenotype, or (F) a CD44^−^ phenotype. Resulting subsets were designated as either stem-like (ALDH^hi^CD44^+^) or non stem-like (ALDH^lo^CD44^−^) and were used immediately for injection into the PuMA.

***Supplementary Fig. 2:*** (A) Schematic showing delivery of breast cancer cells into lung in the ex vivo PUMA model. (B-D) MDA-MB-231, SUM159 and SUM149 breast cancer cells were injected (n = 3 mice). Five random images were taken for each of three lung sections for each time point. A mean normalized fluorescent area (μm2) per image was measured and averaged for each time point.

***Supplementary Fig. 3:*** (A) SUM159 and (B) MDA-MB-231 cells were transfected with either non-targeting (scsi) or *DACH1* targeting (si*DACH1*) siRNA. Significant decrease in DACH1 transcript and protein level is observed at 72h post si*DACH1* transfection as compared to scsi. (C) Loss of function of *DACH1* in MDA-MB-231 resulted in increased acquisition of stem-like ALDH^hi^CD44^+^ phenotype. (D) MDA-MB-231 was exposed to 10ng of recombinant mouse (mFGF2) and human (hFGF2) for 72 hours and stemness was assessed by flow cytometry. Data are presented as mean ± SD, 𝛼 = significantly different than control (p ≤ 0.05; n = 3)

***Supplementary Fig. 4:*** DACH1 protein levels were significantly decreased in ***(G)*** SUM159 and MDA-MB-231 breast cancer cells exposed to 100ng of recombinant human FGF2 for 3 hours. ***(H & I)*** SUM159 breast cancer cells were exposed to BM, tnLCM and tbLCM in the presence or absence of neutralizing FGF2 antibody and Dach1 protein levels were assessed. ***(J)*** Acquisition of stem-like ALDH^hi^CD44^+^ phenotype induced by tbLCM was assessed in the presence of neutralizing FGF2 antibody. Data are presented as the mean ± SD (n = 3).
